# Supplementary material for: Mechanism of molnupiravir-induced SARS-CoV-2 mutagenesis
Source: Nat Struct Mol Biol. 2021 Aug 11;28(9):740–6. doi: 10.1038/s41594-021-00651-0 (PMC8437801; doi:10.1038/s41594-021-00651-0)
Supplement: Supplementary file 1 — Supplementary Tables 1–3 and Supplementary Dataset 1. [file 41594_2021_651_MOESM1_ESM.pdf]

nature portfolio

https://doi.org/10.1038/s41594-021-00651-0

Supplementary information

Mechanism of molnupiravir-induced SARS-CoV-2 mutagenesis

In the format provided by the authors and unedited

Supplementary information

Mechanism of molnupiravir-induced SARS-CoV-2 mutagenesis

Florian Kabinger[1*], Carina Stiller[2*], Jana Schmitzová[1*], Christian Dienemann[1], Goran Kokic[1], Hauke S. Hillen[3,4], Claudia Höbartner[2,#] and Patrick Cramer[1,#]

[1]Max Planck Institute for Biophysical Chemistry, Department of Molecular Biology, Am Fassberg 11, 37077 Göttingen, Germany. [2]Universität Würzburg, Lehrstuhl für Organische Chemie I, Am Hubland, 97074 Würzburg, Germany. [3]University Medical Center Göttingen, Department of Cellular Biochemistry, Humboldtallee 23, 37073 Göttingen, Germany. [4]Max Planck Institute for Biophysical Chemistry, Research Group Structure and Function of Molecular Machines, Am Fassberg 11, 37077 Göttingen, Germany. [*]These authors contributed equally.

Supplementary Table 1: RNA substrates

RNA oligonucleotide	Sequence
Product	/56-FAM/rUrGrA rGrCrC rUrArC rGrCrA rGrU
Template 1	5´rUrUrU rArArC rUrGrC rGrUrA ddC 3´
Template 2	5 rCrCrC rUrArC rUrGrC rGrUrA ddC 3´
Template 3	5´rUrUrU rCrArC rUrGrC rGrUrA ddC 3´
Template 4	5´rUrUrU rGrArC rUrGrC rGrUrA ddC 3´
Template 5	5´rUrUrU MrArC rUrGrC rGrUrA ddC 3´
Template 6 (structure)	5´rGrGrG MrArC rUrGrC rGrUrA-3´
RNA-hairpin duplex (structure)	5´rGrCrU rCrArU rArCrC rGrUrA rUrUrG rArGrA rCrCrU rUrUrU rGrGrU rCrUrC rArArU rArCrG rGrUrA rUrGrA rGrCrC rUrArC rGrCrA rGrUrA 3´
RNA-hairpin duplex (structure)	5´rGrCrU rCrArU rArCrC rGrUrA rUrUrG rArGrA rCrCrU rUrUrU rGrGrU rCrUrC rArArU rArCrG rGrUrA rUrGrA rGrCrC rUrArC rGrCrA rGrUrG 3´
RNA-hairpin duplex	/56-FAM/rUrUrU rUrCrA rUrGrC rArCrC rGrCrG rUrArG rUrUrU rUrCrU rArCrG rCrG-3´

Supplementary Table 2. Thermal melting analysis

sequence R – Y	5’CACUGCGUAGR[a] 3’GUGACGCAUCYGAGU5’	5’CACUGCGUAGRCUCA3’ 3’GUGACGCAUCYGAGU5’	5’CAYUGCGUAGGCUCA3’ 3’GURACGCAUCCGAGU5’
G – C	**64.7 °C**	**77.6 °C**	**77.6 °C**
G – M	**61.2 °C**	**71.2 °C**	**73.2 °C**
A – M	**60.6 °C**	**68.2 °C**	**73.8 °C**

[a] R = G or A, Y = C or M

Supplementary Table 3. Sequences and high-resolution ESI-MS data of NHC/M-modified RNA oligonucleotides

Name	5'-sequence-3'	nt	formula	Mass calc.	Mass found
**M_T**	GGGMACUGCGUAp	12	$C_{115}H_{144}N_{48}O_{86}P_{12}$	3944.5221	3944.5297
**M_1**	MACUGCGUAGGCUCA	15	$C_{142}H_{178}N_{56}O_{104}P_{14}$	4764.6688	4764.6760
**M_3**	CAMUGCGUAGGCUCA	15	$C_{142}H_{178}N_{56}O_{104}P_{14}$	4764.6688	4764.6797
**M_5**	UGAGMCUACGCAGUG	15	$C_{143}H_{178}N_{58}O_{104}P_{14}$	4804.6749	4804.6684

Supplementary Data Set 1: NMR spectra of synthetic compounds

5'-*O*-(4,4'-Dimethoxytrityl)-*N*^{4}-hydroxy-2'-*O*-(triisopropylsilyloxy)methylcytidine (compound 1)

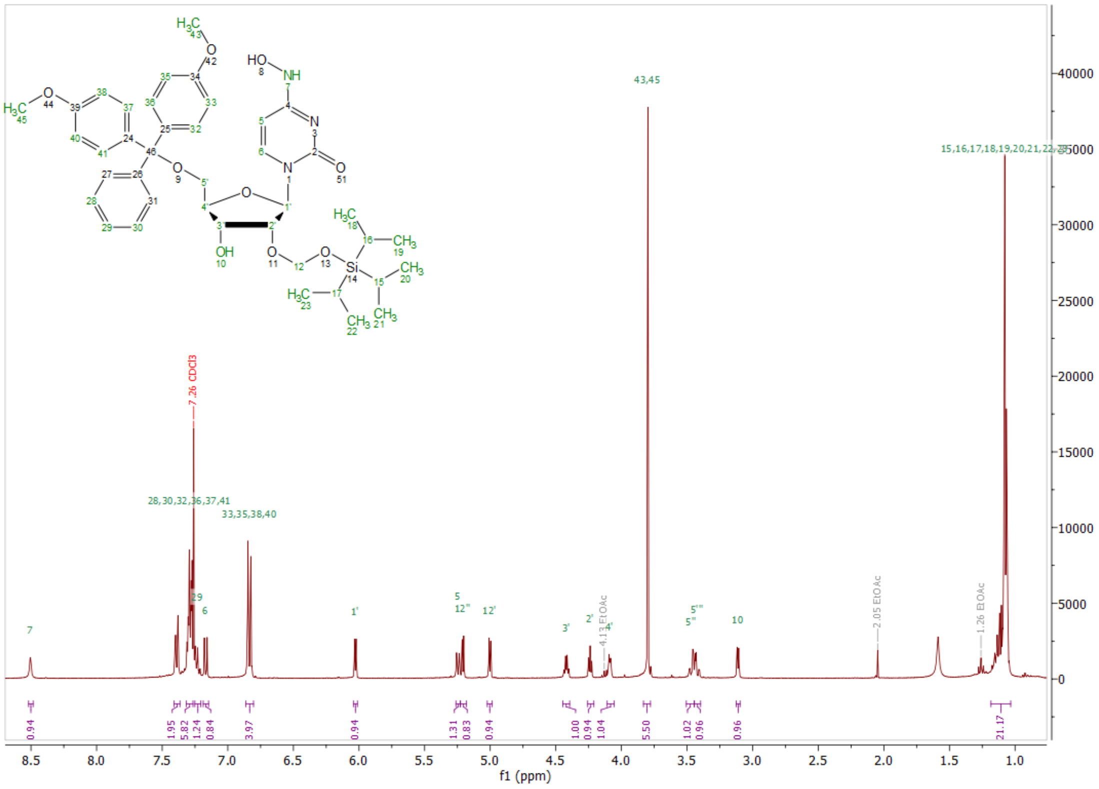

^{1}H-NMR (400 MHz, $CDCl_3$) of compound 1.

^{1}H NMR (400 MHz, $CDCl_3$): δ (ppm) = 1.04 – 1.15 (m, 21H, $Si(CH(CH_3)_2)_3$), 3.13 (d, *J* = 4.7 Hz, 1H, C3'-O*H*), 3.40 – 3.45 (dd, *J* = 2.8, 10.8 Hz, 1H, H-5'b), 3.45 – 3.49 (dd, *J* = 2.4, 10.8 Hz, 1H, H-5'a), 3.80 (s, 6H, OCH_3), 4.07 – 4.11 (dt, *J* = 2.6, 5.1 Hz, 1H, H-4'), 4.24 (t, *J* = 4.8 Hz, 1H, H-2'), 4.38 – 4.46 (q, *J* = 4.8 Hz, 1H, H-3'), 5.00 (d, *J* = 4.8 Hz, 1H, OCH_2O), 5.20 (d, *J* = 4.8 Hz, 1H, OCH_2O), 5.25 (d, *J* = 8.3 Hz, 1H, H-5), 6.03 (d, *J* = 4.4 Hz, 1H, H-1'), 6.79 – 6.88 (m, 4H, DMT), 7.17 (d, *J* = 8.3 Hz, 1H, H-6), 7.18 – 7.27 (m, 1H, DMT), 7.24 – 7.33 (m, 6H, DMT), 7.35 – 7.42 (m, 2H, DMT), 8.53 (s, NH).

HR-MS (ESI$^+$): Exact mass calculated for $C_{40}H_{53}NaN_3O_9Si$ $[M+Na]^+$: 770.3443, found: 770.3459.

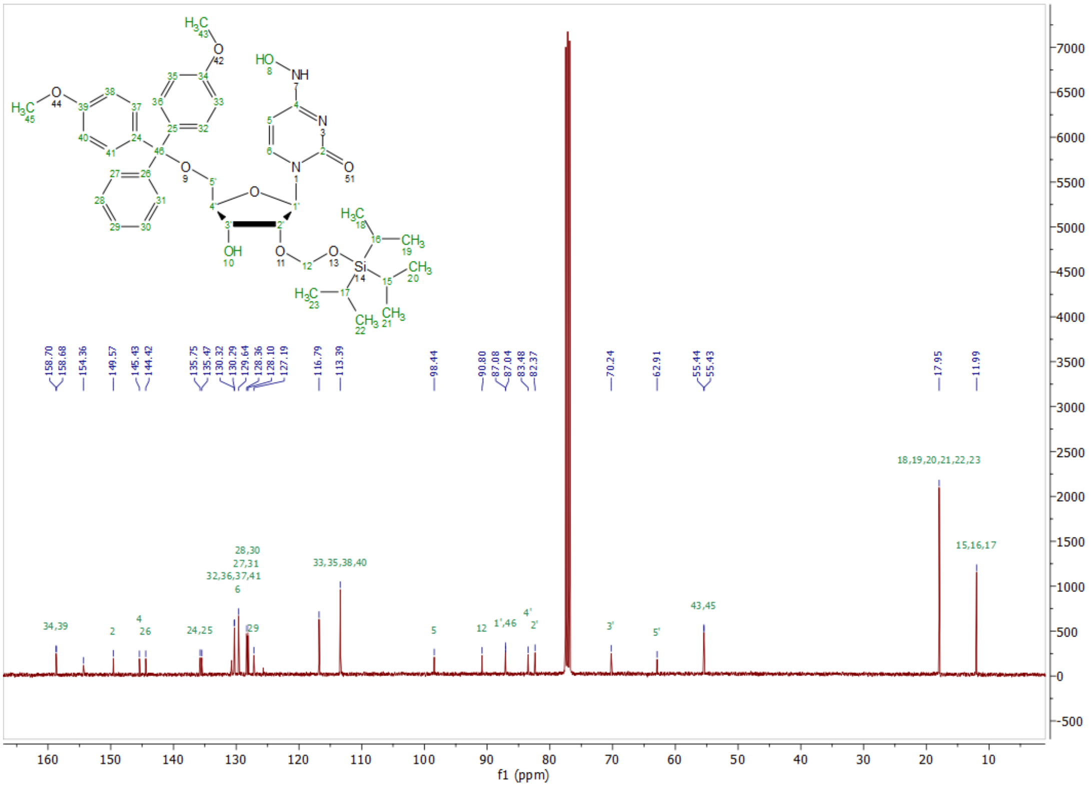

^{13}C-NMR (100 MHz, CDCl$_3$) of compound 1.

^{13}C-NMR (100 MHz, CDCl$_3$): δ (ppm) = 11.99, 17.95 (Si(CH(CH$_3$)$_2$)$_3$), 55.43, 55.44 (OCH$_3$), 62.91 (C5'), 70.24 (C3'), 82.37 (C2'), 83.48 (C4'), 87.04, 87.08 (C1', C$_q$-DMT), 90.80 (OCH$_2$O), 98.44 (C5), 113.39 (DMT), 127.19 (DMT), 128.10 (DMT), 128.36 (DMT), 129.64 (C6), 130.29, 130.32 (DMT), 135.47 135.75 (C$_q$-DMT), 144.42 (C$_q$-DMT), 145.43 (C4), 149.57 (C2), 158.68, 158.70 (C$_q$-OCH$_3$).

N^4-Benzoyl-5'-*O*-(4,4'-dimethoxytrityl)-N^4-hydroxy-2'-*O*-(triisopropylsilyloxy)methyl cytidine (compound 2)

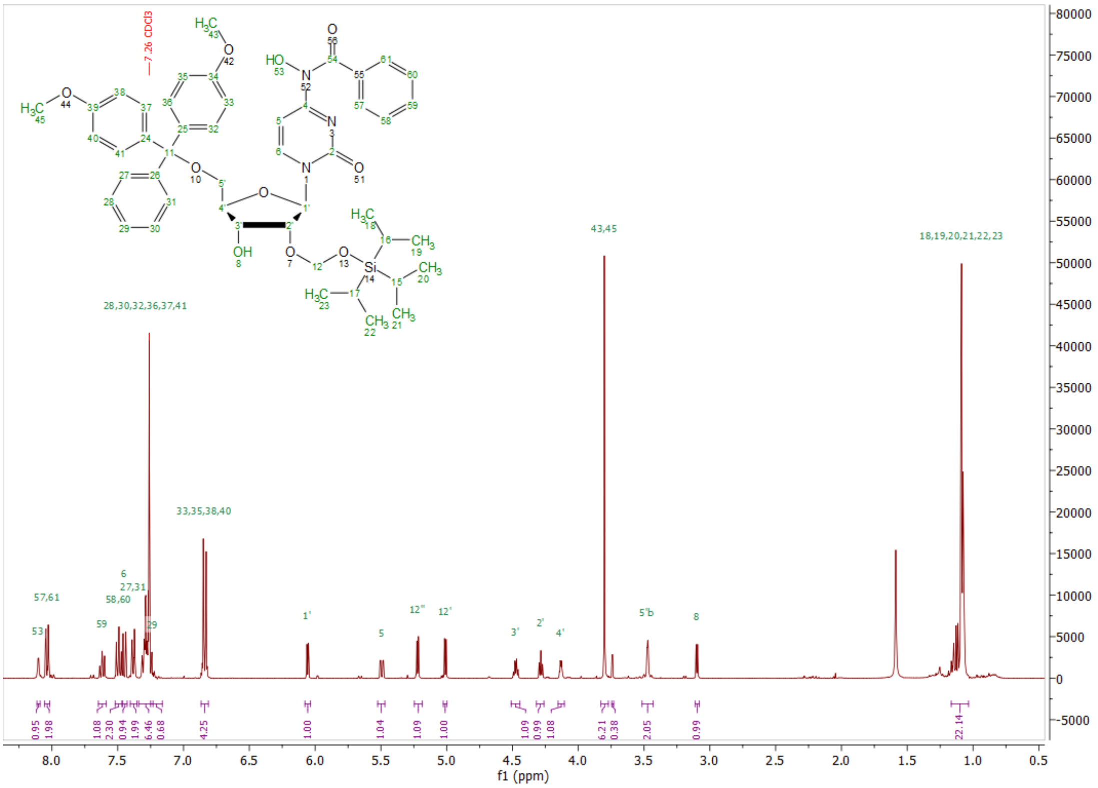

^{1}H-NMR (400 MHz, $CDCl_3$) of compound 2.

^{1}H NMR (400 MHz, $CDCl_3$): δ (ppm) = 1.05 – 1.10 (m, 21H, Si(CH(CH_3)$_2$)$_3$), 3.10 (d, *J* = 4.4 Hz, 1H, C3'-O*H*), 3.44 – 3.50 (m, 2H, H-5'), 3.80 (2s, 6H, OCH_3), 4.11 – 4.16 (m, 1H, H-4'), 4.28 (t, *J* = 4.9 Hz, 1H, H-2'), 4.48 (q, *J* = 4.6 Hz, 1H, H-3'), 5.01 (d, *J* = 4.8 Hz, 1H, OCH_2O), 5.22 (d, *J* = 4.8 Hz, 1H, OCH_2O), 5.49 (dd, *J* = 2.3, 8.3 Hz, 1H, H-5), 6.06 (d, *J* = 4.7 Hz, 1H, H-1'), 6.81 – 6.86 (m, 4H, DMT), 7.22 – 7.34 (m, 6H, DMT), 7.36 – 7.40 (m, 2H,DMT), 7.45 (d, *J* = 8.3 Hz, 1H, H-6), 7.48 – 7.53 (m, 2H, bz), 7.60 – 7.64 (m, 1H, bz), 8.02 – 8.05 (m, 2H, bz), 8.10 (d, *J* = 2.2 Hz, 1H, OH).

HR-MS (ESI$^+$): Exact mass calculated for $C_{47}H_{57}N_3NaO_{10}Si$ [M+Na]$^+$: 874.3705, found: 874.3727.

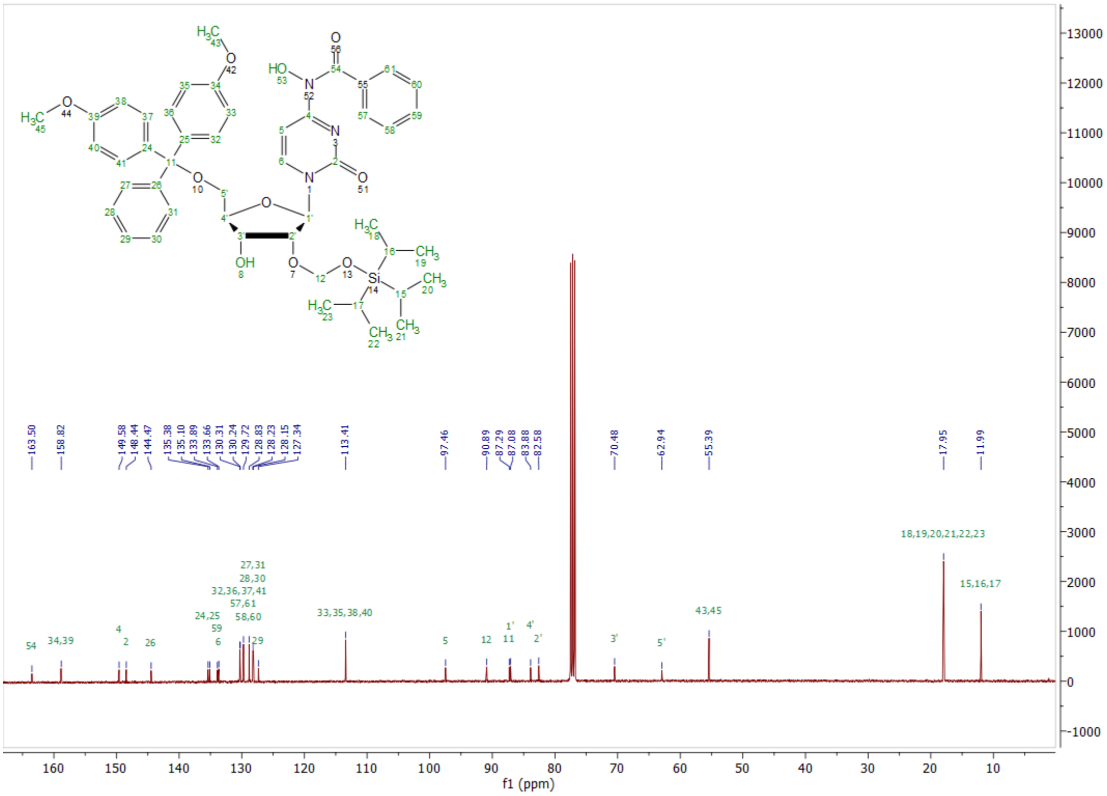

^{13}C-NMR (100 MHz, $CDCl_3$) of compound 2.

^{13}C- NMR (100 MHz, $CDCl_3$): δ (ppm) = 11.99, 17.95 ($Si(CH(CH_3)_2)_3$), 55.39 (OCH_3), 62.94 (C5'), 70.48 (C3'), 82.58 (2'), 83.88 (4'), 87.08 (1'), 87.29 (C_q-DMT), 90.89 (OCH_2O), 97.46 (C5), 113.41 (DMT), 127.34 (DMT), 128.15, 128.23 (DMT), 128.83 (bz), 129.72 (bz), 130.24, 130.31 (DMT), 130.66 (C6), 133.89 (bz), 135.10, 135.38 (C_q-DMT), 144.47 (C_q-DMT), 1448.44 (C2), 149.58 (C4), 158.82 (C_q-OCH_3), 163.50 (C=O).

N^4-Benzoyl-5'-*O*-(4,4'-dimethoxytrityl)-N^4-hydroxy-2'-*O*-(triisopropylsilyloxy)methyl cytidine 3'-cyanoethyl-*N*,*N*-diisopropylphosphoramidite (compound 3, M-PA)

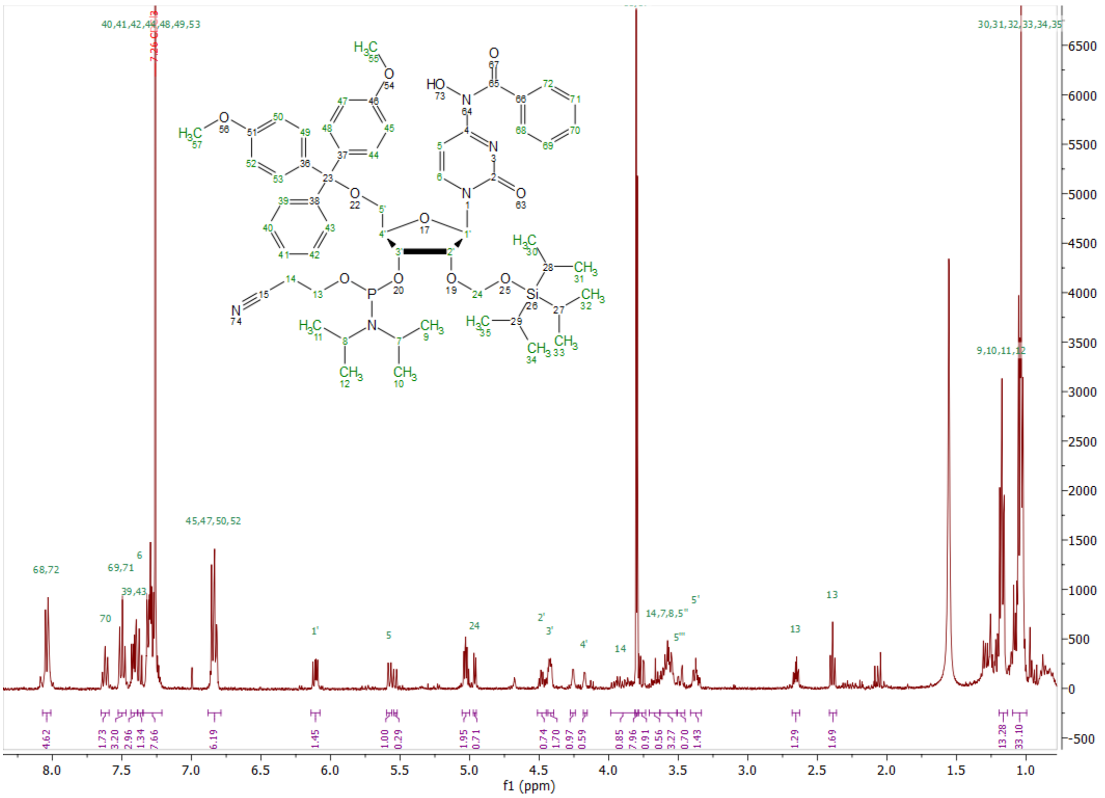

^{1}H-NMR (400 MHz, $CDCl_3$) of compound 3, M-PA.

^{1}H NMR (400 MHz, $CDCl_3$): δ (ppm) = 1.00 – 1.10 (m, 33H, $Si(CH(CH_3)_2)_3$, diast.), 1.13 – 1.19 (m, 13H, $NCH(C\mathit{H}_3)_2$), 2.36 – 2.41 (t, *J* = 6.4 Hz, 2H, $POCH_2$), 2.63 – 2.68 (td, *J* = 2.9, 6.4 Hz, 1H, $POCH_2$, diast.), 3.33 – 3.41 (m, 1H, H-5', diast.), 3.45 – 3.51 (m, 1H, H-5', diast.), 3.51 – 3.63 (m, 3H, H-5', $NC\mathit{H}(CH_3)_2$, CH_2-CN), 3.63 – 3.71 (m, 1H, CH_2-CN, diast.), 3.79 – 3.81 (s, 6H, OCH_3), 3.81 – 3.99 (m, 1H, CH_2-CN), 4.15 – 4.18 (m, 1H, H-4', diast.), 4.24 – 4.28 (m, 1H, H-4'), 4.39 – 4.44 (m, 2H, H-2', H-3'), 4.45 – 4.51 (q, *J* = 5.6 Hz, 1H, H-2'), 4.96 (d, *J* = 5.0 Hz, 1H, OCH_2O, diast.), 5.00 – 5.05 (m, 2H, OCH_2O), 5.54 (d, *J* = 8.2 Hz, 1H, C-5, diast.), 5.58 (d, *J* = 8.2 Hz, 1H, C-5, diast.), 6.08 – 6.14 (2d, *J* = 8.2 Hz, 1H, H-1'), 6.79 – 6.88 (m, 6H, DMT), 7.21 – 7.35 (m, 8H, DMT), 7.35 – 7.39 (m, 1H, H-6), 7.39 – 7.44 (m, 3H, DMT), 7.47 – 7.53 (m, 3H, bz), 7.59 – 7.65 (m, 2H, bz), 8.03 (m, 5H, bz).

HR-MS (ESI$^+$): Exact mass calculated for $C_{56}H_{75}N_5O_{11}PSi$ $[M+H]^+$: 1052.4964, found: 1052.4933 and for $C_{56}H_{74}N_5NaO_{11}PSi$ $[M+Na]^+$: 1074.4784, found: 1074.4749

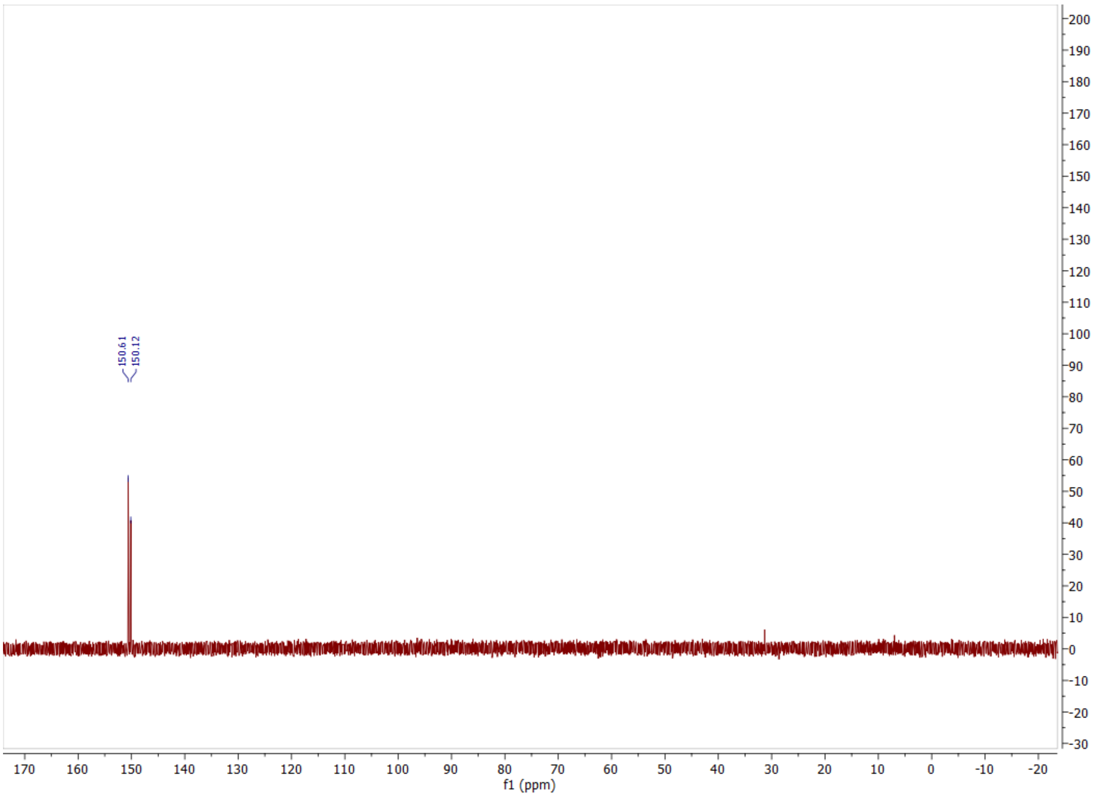

^{31}P-NMR (162 MHz, $CDCl_3$) of compound 3, M-PA.

^{31}P NMR (162 MHz, $CDCl_3$): *δ* (ppm) = 150.12, 150.61.
