# Supplementary material for: Mechanism of molnupiravir-induced SARS-CoV-2 mutagenesis
Source: Nat Struct Mol Biol. 2021 Aug 11;28(9):740–6. doi: 10.1038/s41594-021-00651-0 (PMC8437801; doi:10.1038/s41594-021-00651-0)
Supplement: Supplementary file 2 — Reporting Summary [file 41594_2021_651_MOESM2_ESM.pdf]

nature research

Corresponding author(s): Patrick Cramer; Claudia Höbartner

Last updated by author(s): Jul 26, 2021

Reporting Summary

Nature Research wishes to improve the reproducibility of the work that we publish. This form provides structure for consistency and transparency in reporting. For further information on Nature Research policies, see our Editorial Policies and the Editorial Policy Checklist.

Statistics

For all statistical analyses, confirm that the following items are present in the figure legend, table legend, main text, or Methods section.

n/a	Confirmed	
☐	☒	The exact sample size (n) for each experimental group/condition, given as a discrete number and unit of measurement
☒	☐	A statement on whether measurements were taken from distinct samples or whether the same sample was measured repeatedly
☒	☐	The statistical test(s) used AND whether they are one- or two-sided<br>*Only common tests should be described solely by name; describe more complex techniques in the Methods section.*
☒	☐	A description of all covariates tested
☒	☐	A description of any assumptions or corrections, such as tests of normality and adjustment for multiple comparisons
☐	☒	A full description of the statistical parameters including central tendency (e.g. means) or other basic estimates (e.g. regression coefficient) AND variation (e.g. standard deviation) or associated estimates of uncertainty (e.g. confidence intervals)
☒	☐	For null hypothesis testing, the test statistic (e.g. F, t, r) with confidence intervals, effect sizes, degrees of freedom and P value noted<br>*Give P values as exact values whenever suitable.*
☒	☐	For Bayesian analysis, information on the choice of priors and Markov chain Monte Carlo settings
☒	☐	For hierarchical and complex designs, identification of the appropriate level for tests and full reporting of outcomes
☒	☐	Estimates of effect sizes (e.g. Cohen's d, Pearson's r), indicating how they were calculated

Our web collection on statistics for biologists contains articles on many of the points above.

Software and code

Policy information about availability of computer code

Data collection	Serial EM 3.8 beta 8
Data analysis	RELION 3.1, USCF ChimeraX v0.8, Pymol 2.2.2, Coot 0.9, Warp v1.0.9, PHENIX 1.18, crypSPARC 2.15, Prism 9, Biorad Image Lab v6.1

For manuscripts utilizing custom algorithms or software that are central to the research but not yet described in published literature, software must be made available to editors and reviewers. We strongly encourage code deposition in a community repository (e.g. GitHub). See the Nature Research guidelines for submitting code & software for further information.

Data

Policy information about availability of data

All manuscripts must include a data availability statement. This statement should provide the following information, where applicable:

- Accession codes, unique identifiers, or web links for publicly available datasets
- A list of figures that have associated raw data
- A description of any restrictions on data availability

The cryo-EM reconstructions and structure coordinates for the RdRp-RNA structures containing M–A or M–G base pairs were deposited with the Electron Microscopy Database (EMDB) under accession codes EMD-13135 and EMD-13138 and with the Protein Data Bank (PDB) under accession codes 7OZU and 7OZV, respectively. Source data are provided with this paper. Other data are available from corresponding authors upon reasonable request.

Field-specific reporting

Please select the one below that is the best fit for your research. If you are not sure, read the appropriate sections before making your selection.

☒ Life sciences ☐ Behavioural & social sciences ☐ Ecological, evolutionary & environmental sciences

For a reference copy of the document with all sections, see nature.com/documents/nr-reporting-summary-flat.pdf

Life sciences study design

All studies must disclose on these points even when the disclosure is negative.

Sample size	No statistical methods were used to predetermine sample size. For cryo-EM samples, nine grids of each RdRp-RNA complex (M-G and M-A) were pre-screened to identify the optimal grid for data collection. The number of grids screened was random and was not limited by any experimental parameter.
Data exclusions	No data were excluded from the analyses.
Replication	All attempts of replication were succesful. Cryo-EM single particle analysis inherently relies on averaging a large number of independent observations. All biochemical experiments that were quantified were performed in independent triplicates. Results shown in figure 2b and 2d were performed once under exact same conditions.
Randomization	Samples were not allocated to groups. All cryo-EM particles used for structure determination adopt random orientations in the ice on the grid. Division of particles into random halves was automatically performed during 3D reconstruction by Relion 3.1. Other experiments did not involve randomization
Blinding	Blinding is not applicable for this study, as group allocation is not used.

Reporting for specific materials, systems and methods

We require information from authors about some types of materials, experimental systems and methods used in many studies. Here, indicate whether each material, system or method listed is relevant to your study. If you are not sure if a list item applies to your research, read the appropriate section before selecting a response.

Materials & experimental systems

n/a	Involved in the study	
☒	☐	Antibodies
☐	☒	Eukaryotic cell lines
☒	☐	Palaeontology and archaeology
☒	☐	Animals and other organisms
☒	☐	Human research participants
☒	☐	Clinical data
☒	☐	Dual use research of concern

Methods

n/a	Involved in the study	
☒	☐	ChIP-seq
☒	☐	Flow cytometry
☒	☐	MRI-based neuroimaging

Eukaryotic cell lines

Policy information about cell lines

Cell line source(s)	Hi 5 cells: Expression System, Tni Insect cells in ESF921 media
Authentication	None of the cell lines were authenticated.
Mycoplasma contamination	Cell lines were not tested for mycoplasma contamination.
Commonly misidentified lines (See ICLAC register)	No commonly misidentified cell lines were used.
