# Supplementary material for: Mechanism of molnupiravir-induced SARS-CoV-2 mutagenesis
Source: Nat Struct Mol Biol. 2021 Aug 11;28(9):740–6. doi: 10.1038/s41594-021-00651-0 (PMC8437801; doi:10.1038/s41594-021-00651-0)
Supplement: Supplementary file 3 — Peer Review Information [file 41594_2021_651_MOESM3_ESM.pdf]

Peer Review Information

Journal: Nature Structural and Molecular Biology
Manuscript Title: Mechanism of molnupiravir-induced SARS-CoV-2 mutagenesis
Corresponding author name(s): Patrick Cramer

Reviewer Comments & Decisions:

Decision Letter, initial version:

23rd Jun 2021

Dear Patrick,

Thank you again for submitting your manuscript "Mechanism of molnupiravir-induced SARS-CoV-2 mutagenesis". I apologize for the delay while we awaited the comments (copied below) from the 3 reviewers who evaluated your paper. In light of those reports, we remain interested in your study and would like to see your response to the comments of the referees, in the form of a revised manuscript.

You will see that Reviewers 2 & 3 both note that the biochemical analysis of Molnupiravir activity has been reported in a recent publication by Götte and colleagues (doi: 10.1016/j.jbc.2021.100770) that needs to be cited and discussed in a potential revised manuscript. While Reviewer #2 is not in favor of publication in NSMB, editorially, we agree with Reviewer #3 that the additional structure is of sufficient value to inform antiviral drug design to consider the work further for publication in our journal. Reviewer #1 queries several aspects of the biochemical analysis that remain to be addressed within the text. Please be sure to address/respond to all concerns of the referees in full in a point-by-point response and highlight all changes in the revised manuscript text file. If you have comments that are intended for editors only, please include those in a separate cover letter.

We are committed to providing a fair and constructive peer-review process. Do not hesitate to contact us if there are specific requests from the reviewers that you believe are technically impossible or unlikely to yield a meaningful outcome.

We expect to see your revised manuscript within 3 weeks. If you cannot send it within this time, please contact us to discuss an extension; we would still consider your revision, provided that no similar work has been accepted for publication at NSMB or published elsewhere.

As you already know, we put great emphasis on ensuring that the methods and statistics reported in our papers are correct and accurate. As such, if there are any changes that should be reported, please

submit an updated version of the Reporting Summary along with your revision.

Please follow the links below to download these files:

Reporting Summary:
https://www.nature.com/documents/nr-reporting-summary.pdf

Please note that the form is a dynamic 'smart pdf' and must therefore be downloaded and completed in Adobe Reader.

When submitting the revised version of your manuscript, please pay close attention to our href="https://www.nature.com/nature-research/editorial-policies/image-integrity">Digital Image Integrity Guidelines. and to the following points below:

-- that unprocessed scans are clearly labelled and match the gels and western blots presented in figures.
-- that control panels for gels and western blots are appropriately described as loading on sample processing controls
-- all images in the paper are checked for duplication of panels and for splicing of gel lanes.

Finally, please ensure that you retain unprocessed data and metadata files after publication, ideally archiving data in perpetuity, as these may be requested during the peer review and production process or after publication if any issues arise.

If there are additional or modified structures presented in the final revision, please submit the corresponding PDB validation reports.

Please note that all key data shown in the main figures as cropped gels or blots should be presented in uncropped form, with molecular weight markers. These data can be aggregated into a single supplementary figure item. While these data can be displayed in a relatively informal style, they must refer back to the relevant figures. These data should be submitted with the final revision, as source data, prior to acceptance, but you may want to start putting it together at this point.

SOURCE DATA: we urge authors to provide, in tabular form, the data underlying the graphical representations used in figures. This is to further increase transparency in data reporting, as detailed in this editorial (http://www.nature.com/nsmb/journal/v22/n10/full/nsmb.3110.html). Spreadsheets can be submitted in excel format. Only one (1) file per figure is permitted; thus, for multi-paneled figures, the source data for each panel should be clearly labeled in the Excel file; alternately the data can be provided as multiple, clearly labeled sheets in an Excel file. When submitting files, the title field should indicate which figure the source data pertains to. We encourage our authors to provide source data at the revision stage, so that they are part of the peer-review process.

Data availability: this journal strongly supports public availability of data. All data used in accepted papers should be available via a public data repository, or alternatively, as Supplementary Information. If data can only be shared on request, please explain why in your Data Availability Statement, and also in the correspondence with your editor. Please note that for some data types,

deposition in a public repository is mandatory - more information on our data deposition policies and available repositories can be found below:
https://www.nature.com/nature-research/editorial-policies/reporting-standards#availability-of-data

We require deposition of coordinates (and, in the case of crystal structures, structure factors) into the Protein Data Bank with the designation of immediate release upon publication (HPUB). Electron microscopy-derived density maps and coordinate data must be deposited in EMDB and released upon publication. Deposition and immediate release of NMR chemical shift assignments are highly encouraged. Deposition of deep sequencing and microarray data is mandatory, and the datasets must be released prior to or upon publication. To avoid delays in publication, dataset accession numbers must be supplied with the final accepted manuscript and appropriate release dates must be indicated at the galley proof stage.

While we encourage the use of color in preparing figures, please note that this will incur a charge to partially defray the cost of printing. Information about color charges can be found at
http://www.nature.com/nsmb/authors/submit/index.html#costs

Please use the link below to submit your revised manuscript and related files:

[REDACTED]

Note: This URL links to your confidential home page and associated information about manuscripts you may have submitted, or that you are reviewing for us. If you wish to forward this email to co-authors, please delete the link to your homepage.

We look forward to seeing the revised manuscript and thank you for the opportunity to review your work.

With kind regards,

Beth

Beth Moorefield, Ph.D.
Senior Editor
Nature Structural & Molecular Biology

Referee expertise:

Referee #1: RNAP inhibitors

Referee #2: viral transcription mechanisms

Referee #3: structural biology

Reviewers' Comments:

Reviewer #1:
Remarks to the Author:
In the submitted manuscript the Cramer and Höbartner groups investigate the action of molnupiravir (M) on SARS-CoV-2 RNA-dependent RNA polymerase (RdRp). The compound is known to inhibit viral replication and is currently in clinical trials for treatment of Covid-19 patients. The authors provide biochemical as well structural data that corroborate earlier assumptions on the mode of action of M. The authors nicely show that indeed M is introducing errors at high frequency during viral replication presumably leading to the error-catastrophe which will result in inhibition of viral replication.

Obviously, the topic is very timely. The structural data is of high quality. However, in my eyes the biochemical analysis is too immature and requires some additional experiments / discussion:

a) In their biochemical experiments the authors used different RNA duplexes. In my eyes these complexes are somehow awkward: Why does the primer have a single stranded overhang towards the 5'-end? Why does the template strand bears a mispaired nucleotide at the 3'-end? This is puzzling and need some explanation and argumentation why these constructs were used. Furthermore, these constructs are different from those used in earlier studies of the group. Again, this need some explanation Also, it needs to be discussed whether they represent the natural circumstances of viral replication.
b) The authors do not provide any information on how the standard deviations were determined in their RNA extension assays. Where the experiments repeated (which I judge being essential)? If yes, how many times?
c) Kinetic data such as KM and kcat values are needed to quantify the observed effects. This would significantly increase the significance of the study and allow to address important questions such as: what is the incorporation efficiency of M and opposite M in the template in comparison canonical substrates? What is the extension from M in the primer in comparison canonical substrates?
One other issue needs to be addressed: To be a suitable antiviral drug acting on a nucleic acid polymerase, a compound needs to fulfil two criteria: It has to "interfere" with the viral enzyme but should not affect the host's enzymes. The later issue is completely ignored in the manuscript. Is M a poor substrate for the human RNA polymerases? At least some discussion (better: results) along these lines are needed.

After these issues have been addressed in an appropriate fashion, the manuscript will make a fine contribution to Nature SMB.

Reviewer #2:
Remarks to the Author:
In "Mechanism of molnupiravir-induced SARS-CoV-2 mutagenesis", Kabinger and colleagues describe the mechanism of SARS-CoV-2 replication inhibition by Molnupiravir, a broad-spectrum mutagenic nucleotide analogue that induces an error catastrophe in the product strand. They provide biochemical and structural data to show that Molnupiravir is well incorporated by SARS-CoV-2 polymerase opposite to A or G without inducing stalling or termination. Furthermore, they show that SARS-CoV-2 polymerase utilizes RNA substrate containing Molnupiravir (M) to incorporate A or G with equivalent efficiency. Last, they provide structural information to show M:A and M:G interactions with M in the template strand.

While the study is well performed and the paper well written, the biochemical work is not new and was recently published by Gordon et al., JBC 2021 (https://doi.org/10.1016/j.jbc.2021.100770), which includes a more in-depth kinetic study than the one presented here. Furthermore, the structural work seems at odd with their previous remarkable cryoEM structure of the SARS-CoV-2 elongation complex (Hillen et al, 2020), as they could not resolve the nsp8 "pols", and does not provide essential information to understand the mechanism of action of Molnupiravir.

This work is therefore not novel (biochemistry) and groundbreaking enough (structure) to warrant publication in Nature Structural and Molecular Biology, and I therefore don't recommend publication.

Reviewer #3:
Remarks to the Author:
The Hobartner and Cramer groups have addressed the biochemical and structural basis of Molnupiravir (M) activity as a mutagenic nucleoside analog. Molnupiravir is an important broad-spectrum antiviral that shows potent activity against SARS-CoV-2 and thus is being investigated as a therapeutic against covid-19 infections. In addition, the mechanism of this class of antivirals that works by lethal mutagenesis has only recently been explored. Therefore, the significance of this study is high. The experimental design and results are rigorously presented, and the manuscript is well-written.
The research presented here shows:
1)M is incorporated opposite G and A templates, with a preference for G.
2) However, that incorporation does not cause stalling or termination of downstream NTP incorporation.
3) Rather, it appears that when M is in the template RNA, it incorporates A and G, again with a preference for G but with significantly decreased selectivity.
4) Thus, the increased promiscuity of M as a template is the basis of its mutagenic activity.
5) The authors then show the structural basis for M as a template for both G and A in its two tautomeric forms.

The main detraction of this manuscript is that much of the biochemistry has been previously reported in a manuscript recently published online (May 10) by the Götte Lab in JBC. Gotte has shown almost the exact mechanism with similar experiments as those presented in figures 1-3. This new manuscript should now be added and discussed in a revised version https://www.jbc.org/article/S0021-9258(21)00563-9/fulltext. That said, the structural basis is always a welcome addition as it confirms the base-pairing previously proposed and offers a platform for rational drug design. I also think that confirmatory studies are essential in this field, especially by such rigorous studies like the one presented here. Having both the biochemical and structural basis of lethal mutagenesis has been a significant gap in understanding this class of antivirals, so the work here is highly impactful. The authors could also put some of the RNA synthesis data in the main figures.

Minor points to address are:
1) The discussion on the difference in the selectivity of M as a substrate versus a template is lacking. Why does it significantly prefer base-pairing with G over A as a substrate versus a template?
2) How do the authors reconcile the different tautomers of M and their structures? Are each (M-A and M-G) a single but distinct tautomer when base-paired with G or A?
3) Can the authors discuss their results in the context of the observed mutagenic patterns observed in vivo?

4) Can the authors calculate a 3D-FSC? While the maps are impressive in the active site, it does appear there is severe particle orientation bias, and it would be good to have this analysis.
5) Lines 76-78 "Time-dependent RNA elongation experiments showed that M was slightly less efficiently incorporated as the cognate nucleotide C, but less efficiently than the cognate nucleotide U" is a bit unclear. I know what the authors are trying to say, but it can be stated more clearly.

Author Rebuttal to Initial comments

Responses to reviewer concerns
Kabinger et al. (NSMB-A44955)

Responses are in italics

Reviewer #1:

In the submitted manuscript the Cramer and Höbartner groups investigate the action of molnupiravir (M) on SARS-CoV-2 RNA-dependent RNA polymerase (RdRp). The compound is known to inhibit viral replication and is currently in clinical trials for treatment of Covid-19 patients. The authors provide biochemical as well structural data that corroborate earlier assumptions on the mode of action of M. The authors nicely show that indeed M is introducing errors at high frequency during viral replication presumably leading to the error-catastrophe which will result in inhibition of viral replication.

We thank the reviewer for the kind words and useful comments.

Obviously, the topic is very timely. The structural data is of high quality. However, in my eyes the biochemical analysis is too immature and requires some additional experiments / discussion:

a) In their biochemical experiments the authors used different RNA duplexes. In my eyes these complexes are somehow awkward: Why does the primer have a single stranded overhang towards the 5'-end? Why does the template strand bears a mispaired nucleotide at the 3'-end?
This is puzzling and need some explanation and argumentation why these constructs were used.

Furthermore, these constructs are different from those used in earlier studies of the group. Again, this need some explanation Also, it needs to be discussed whether they represent the natural circumstances of viral replication.

We have added several sentences to the Methods section to explain in detail the reasons for this design of RNA scaffolds. We also explain why these constructs differ from previously used ones (lines 510 - 517).

b) The authors do not provide any information on how the standard deviations were determined in their RNA extension assays. Where the experiments repeated (which I judge being essential)? If yes, how many times?

We have added the information on replicates and standard deviations to the Methods and figure legends (figure legends and lines 531 – 533).

c) Kinetic data such as KM and kcat values are needed to quantify the observed effects. This would significantly increase the significance of the study and allow to address important questions such as: what is the incorporation efficiency of M and opposite M in the template in comparison canonical substrates? What is the extension from M in the primer in comparison canonical substrates?

From the available data, we cannot infer Michaelis-Menten parameters; this would require additional experiments. We however note that we added emphasis on existing biochemical data that addresses important aspects. In particular, we added a sentence to the results section describing the incorporation efficiency of M opposite of templating G and A, in more detail (figure panels 1f and 1g showing time-dependent M incorporation opposite templating G or A). Moreover, we also describe the extension from M in the product strand in comparison to canonical substrates and show that M incorporation does not stall RdRp (Fig. 2). Overall, we have arrived at a good characterization of the enzymatic properties.

In addition, after the submission of our manuscript, independently derived biochemical data about the Molnupiravir mechanism of action became available that are consistent with our findings. (DOI: 10.1016/j.jbc.2021.100770). The published data confirms all our biochemical results and provide complementary details about steady-state kinetics. We included a sentence about the new manuscript in the end of the discussion.

One other issue needs to be addressed: To be a suitable antiviral drug acting on a nucleic acid polymerase, a compound needs to fulfil two criteria: It has to “interfere” with the viral enzyme but should not affect the host’s enzymes. The later issue is completely ignored in the manuscript. Is M a poor substrate for the human RNA polymerases? At least some discussion (better: results) along these lines are needed.

We have added a short paragraph at the end of the manuscript to address this important point. It is known from the literature that M can be incorporated into RNA by the host mitochondrial RNA polymerase and we have cited this work. The situation for the nuclear RNA polymerases is unclear and should be studied in the future. Such experiments are however beyond the scope of our current manuscript (lines 179 – 184).

After these issues have been addressed in an appropriate fashion, the manuscript will make a fine contribution to Nature SMB.

We thank the reviewer again for the good suggestions and comments.

Reviewer #2:

In "Mechanism of molnupiravir-induced SARS-CoV-2 mutagenesis", Kabinger and colleagues describe the mechanism of SARS-CoV-2 replication inhibition by Molnupiravir, a broad-spectrum mutagenic nucleotide analogue that induces an error catastrophe in the product strand. They provide biochemical and structural data to show that Molnupiravir is well incorporated by SARSCoV-2 polymerase opposite to A or G without inducing stalling or termination. Furthermore, they show that SARS-CoV-2 polymerase utilizes RNA substrate containing Molnupiravir (M) to incorporate A or G with equivalent efficiency. Last, they provide structural information to show M:A and M:G interactions with M in the template strand.

While the study is well performed and the paper well written, the biochemical work is not new and was recently published by Gordon et al., JBC 2021

(https://doi.org/10.1016/j.jbc.2021.100770), which includes a more in-depth kinetic study than the one presented here.

We thank the reviewer for the positive comments. We note that the competing work (Gordon et al.) appeared online on May 11, 2021, the same day that our manuscript was posted on BioXiv online (doi: https://doi.org/10.1101/2021.05.11.443555). Therefore it is clearly documented all our work was performed independently. In addition, our manuscript goes well beyond the work published by Gordon et al., in particular by using synthetic RNA with M at defined positions and by providing the structural basis for M-induced mutagenesis. In summary, it cannot be argued that our work is compromised in novelty due to the competing study. We have however now included this reference in the discussion of our paper in a scholarly manner.

Furthermore, the structural work seems at odd with their previous remarkable cryoEM structure of the SARS-CoV-2 elongation complex (Hillen et al, 2020), as they could not resolve the nsp8 "pols", and does not provide essential information to understand the mechanism of action of Molnupiravir.

*We disagree that our structural work does "*not provide essential information to understand the mechanism of action of Molnupiravir*". In contrast, our structural work shows the formation of Mcontaining base pairs for the first time. It is a big difference to predict the formation of Mcontaining base pairs and to observed such base pairs directly.*

*We also disagree that the structural work "*seems at odd with their previous remarkable cryoEM structure of the SARS-CoV-2 elongation complex (Hillen et al, 2020)*" because, as we have written in the manuscript, the nsp8 poles are flexible but indeed observed here again, as reported in our original paper, but were removed from the modeling and refinement.*

This work is therefore not novel (biochemistry) and groundbreaking enough (structure) to warrant publication in Nature Structural and Molecular Biology, and I therefore don't recommend publication.

We accept that the reviewer has this opinion but wish to reiterate that we believe our work is a highly valuable structure-function analysis with many novel aspects that addresses a biomedically important mechanism in a timely manner.

Reviewer #3:

The Hobartner and Cramer groups have addressed the biochemical and structural basis of Molnupiravir (M) activity as a mutagenic nucleoside analog. Molnupiravir is an important broadspectrum antiviral that shows potent activity against SARS-CoV-2 and thus is being investigated as a therapeutic against covid-19 infections. In addition, the mechanism of this class of antivirals that works by lethal mutagenesis has only recently been explored. Therefore, the significance of this study is high. The experimental design and results are rigorously presented, and the manuscript is well-written.

The research presented here shows:

1)M is incorporated opposite G and A templates, with a preference for G.
2)However, that incorporation does not cause stalling or termination of downstream NTP incorporation.
3)Rather, it appears that when M is in the template RNA, it incorporates A and G, again with a preference for G but with significantly decreased selectivity.

4)Thus, the increased promiscuity of M as a template is the basis of its mutagenic activity. 5) The authors then show the structural basis for M as a template for both G and A in its two tautomeric forms.

We would like to thank the reviewer for the kind words and the important comments that have improved our manuscript.

The main detraction of this manuscript is that much of the biochemistry has been previously reported in a manuscript recently published online (May 10) by the Götte Lab in JBC. Gotte has shown almost the exact mechanism with similar experiments as those presented in figures 1-3. This new manuscript should now be added and discussed in a revised

version https://www.jbc.org/article/S0021-9258(21)00563-9/fulltext. That said, the structural basis is always a welcome addition as it confirms the base-pairing previously proposed and offers a platform for rational drug design. I also think that confirmatory studies are essential in this field, especially by such rigorous studies like the one presented here. Having both the biochemical and structural basis of lethal mutagenesis has been a significant gap in understanding this class of antivirals, so the work here is highly impactful. The authors could also put some of the RNA synthesis data in the main figures.

We very much appreciate the reviewer's comment that our work is important to be published despite the other paper that appeared meanwhile. Indeed the competing work (Gordon et al.) appeared online on May 11, 2021, the same day that our manuscript was posted on BioXiv online (doi: https://doi.org/10.1101/2021.05.11.443555). Therefore it is clearly documented all our work was performed independently. In addition, our manuscript goes well beyond the work published by Gordon et al., in particular by using synthetic RNA with M at defined positions and by providing the structural basis for M-induced mutagenesis. We have included the competing paper as a new reference in the discussion of our manuscript.

Minor points to address are:

1) The discussion on the difference in the selectivity of M as a substrate versus a template is lacking. Why does it significantly prefer base-pairing with G over A as a substrate versus a template?

We assume this is a misunderstanding. We show that in both scenarios, the observed results can be explained by formation of a M-G (or G-M) base pair (for M incorporation see Fig. 1 and for the templating M see Fig. 3. We went through the text again and made sure this is correctly understood.

2) How do the authors reconcile the different tautomers of M and their structures? Are each (M-A and M-G) a single but distinct tautomer when base-paired with G or A?

This is what we assume when we interpret the density but of course we can only infer this from structural data. To address this point, we have added text to the Discussion section to explain the observed EM densities (lines 158 – 160).

3) Can the authors discuss their results in the context of the observed mutagenic patterns observed in vivo?

We had mentioned the in vivo results in the introduction but now also mention them in one additional sentence again in the discussion (lines 160- 162).

4) Can the authors calculate a 3D-FSC? While the maps are impressive in the active site, it does appear there is severe particle orientation bias, and it would be good to have this analysis.

We added the particle orientation information to the manuscript (Extended Data Figure 4 b, d).

5) Lines 76-78 "Time-dependent RNA elongation experiments showed that M was slightly less efficiently incorporated as the cognate nucleotide C, but less efficiently than the cognate nucleotide U" is a bit unclear. I know what the authors are trying to say, but it can be stated more clearly.

To make this clearer, we split these results in two sentences and improved the language (lines 76 – 79). Maybe this was the reason for the misunderstanding (point 1).

Decision Letter, first revision:

15th Jul 2021

Dear Patrick,

Thank you for submitting your revised manuscript "Mechanism of molnupiravir-induced SARS-CoV-2 mutagenesis" (NSMB-A44955A). It has now been seen by one of the original referees and their comments are below. I hope you will be pleased to see that the reviewer finds that the paper has improved in revision, and emphasizes the importance of the study. We'll therefore be happy in principle to publish it in Nature Structural & Molecular Biology, pending minor revisions to comply with our editorial and formatting guidelines.

We are now performing detailed checks on your paper and will send you a checklist detailing our editorial and formatting requirements in a few days. Please do not upload the final materials and make any revisions until you receive this additional information from us.

 To expedite our work at this stage, we would appreciate if you could send us the main text as a Word file as soon as possible. Please make sure to copy the NSMB account (cc'ed above).

We look forward to presenting your study ASAP in Nature Structural & Molecular Biology. Please do not hesitate to contact me if you have any questions.

With kind regards,

Beth

Beth Moorefield, Ph.D.
Senior Editor
Nature Structural & Molecular Biology

Reviewer #3 (Remarks to the Author):

The authors have done an excellent job addressing my initial questions/concerns (which were mostly clarification issues). Visualizing the basis behind lethal mutagenesis will be a significant addition to the field of this class of antivirals. I recommend publishing this important study.

Final Decision Letter:

28th Jul 2021

Dear Patrick,

We are now happy to accept your revised paper "Mechanism of molnupiravir-induced SARS-CoV-2 mutagenesis" for publication as a Article in Nature Structural & Molecular Biology.

Acceptance is conditional on the manuscript's not being published elsewhere and on there being no announcement of this work to the newspapers, magazines, radio or television until the publication date in Nature Structural & Molecular Biology.

Before the manuscript is sent to the printers, we shall make any detailed changes in the text that may be necessary either to make it conform with house style or to make it intelligible to a wider readership. If the changes are extensive, we will ask for your approval before the manuscript is laid out for production. Once your manuscript is typeset you will receive a link to your electronic proof via email within 20 working days, with a request to make any corrections within 48 hours. Please read proofs with great care to make sure that the sense has not been altered. If you have queries at any point during the production process then please contact the production team at rjsproduction@springernature.com. Once your paper has been scheduled for online publication, the

Nature press office will be in touch to confirm the details.

Please note that due to tight production schedules, proofs should be returned as quickly as possible to avoid delaying publication. If you anticipate any limitations to your availability over the next 2-4 weeks (such as vacation or traveling to conferences, etc.), please e-mail rjsproduction@springernature.com as soon as possible. Please provide specific dates that you will be unavailable and provide detailed contact information for an alternate corresponding author if necessary.

To assist our authors in disseminating their research to the broader community, our SharedIt initiative provides all co-authors with the ability to generate a unique shareable link that will allow anyone (with or without a subscription) to read the published article. Recipients of the link with a subscription will also be able to download and print the PDF.

As soon as your article is published, you can generate your shareable link by entering the DOI of your article here: <a href="http://authors.springernature.com/share">http://authors.springernature.com/share<a>. Corresponding authors will also receive an automated email with the shareable link

Note the policy of the journal on data deposition: http://www.nature.com/authors/policies/availability.html.

Your paper will be published online soon after we receive proof corrections and will appear in print in the next available issue. You can find out your date of online publication by contacting the production team shortly after sending your proof corrections. Content is published online weekly on Mondays and Thursdays, and the embargo is set at 16:00 London time (GMT)/11:00 am US Eastern time (EST) on the day of publication. Now is the time to inform your Public Relations or Press Office about your paper, as they might be interested in promoting its publication. This will allow them time to prepare an accurate and satisfactory press release. Include your manuscript tracking number (NSMB-A44955B) and our journal name, which they will need when they contact our press office.

About one week before your paper is published online, we shall be distributing a press release to news organizations worldwide, which may very well include details of your work. We are happy for your institution or funding agency to prepare its own press release, but it must mention the embargo date and Nature Structural & Molecular Biology. If you or your Press Office have any enquiries in the meantime, please contact press@nature.com.

You can now use a single sign-on for all your accounts, view the status of all your manuscript submissions and reviews, access usage statistics for your published articles and download a record of your refereeing activity for the Nature journals.

If you have not already done so, we strongly recommend that you upload the step-by-step protocols used in this manuscript to the Protocol Exchange. Protocol Exchange is an open online resource that allows researchers to share their detailed experimental know-how. All uploaded protocols are made freely available, assigned DOIs for ease of citation and fully searchable through nature.com. Protocols can be linked to any publications in which they are used and will be linked to from your article. You can also establish a dedicated page to collect all your lab Protocols. By uploading your Protocols to

Protocol Exchange, you are enabling researchers to more readily reproduce or adapt the methodology you use, as well as increasing the visibility of your protocols and papers. Upload your Protocols at www.nature.com/protocolexchange/. Further information can be found at www.nature.com/protocolexchange/about.

An online order form for reprints of your paper is available at https://www.nature.com/reprints/author-reprints.html. Please let your coauthors and your institutions' public affairs office know that they are also welcome to order reprints by this method.

Please note that <i>Nature Structural & Molecular Biology</i> is a Transformative Journal (TJ). Authors may publish their research with us through the traditional subscription access route or make their paper immediately open access through payment of an article-processing charge (APC). Authors will not be required to make a final decision about access to their article until it has been accepted. Find out more about Transformative Journals

<B>Authors may need to take specific actions to achieve compliance with funder and institutional open access mandates. For submissions from January 2021, if your research is supported by a funder that requires immediate open access (e.g. according to Plan S principles) then you should select the gold OA route, and we will direct you to the compliant route where possible. For authors selecting the subscription publication route our standard licensing terms will need to be accepted, including our self-archiving policies. Those standard licensing terms will supersede any other terms that the author or any third party may assert apply to any version of the manuscript.

In approximately 10 business days you will receive an email with a link to choose the appropriate publishing options for your paper and our Author Services team will be in touch regarding any additional information that may be required.

You will not receive your proofs until the publishing agreement has been received through our system.

If you have any questions about our publishing options, costs, Open Access requirements, or our legal forms, please contact ASJournals@springernature.com

With kind regards,

Beth

Beth Moorefield, Ph.D.
Senior Editor
Nature Structural & Molecular Biology
